# The efficacy of iCBT added to treatment as usual for alcohol-dependent patients in primary care: study protocol for a randomized controlled trial

**DOI:** 10.1186/s13063-019-3902-6

**Published:** 2019-12-30

**Authors:** Karin Hyland, Anders Hammarberg, Erik Hedman-Lagerlöf, Magnus Johansson, Sven Andreasson

**Affiliations:** 1 Department of Clinical Neuroscience, Karolinska Institutet, 17177 Stockholm, Sweden; 2 Centre for Dependency Disorders, Stockholm Health Care Services, Stockholm County Council, Stockholm, Sweden; 3 Gustavsberg Primary Health Care Center, Stockholm, Sweden; 4 Department of Public Health Sciences, Karolinska Institutet, 17177 Stockholm, Sweden

**Keywords:** Alcohol dependence, RCT, Primary care, Internet-based Cognitive Behavioral Treatment (iCBT)

## Abstract

**Background:**

Alcohol dependence is a common disorder with a continuum regarding severity. Most alcohol-dependent persons have a moderate level of dependence and live under socially orderly conditions. Treatment-seeking in this group is low, mainly due to stigma and because treatment options are seen as unappealing.

Alcohol is a relevant topic to discuss in many primary care (PC) consultations and PC is less stigmatizing to visit compared to addiction care units for people with alcohol problems. However, general practitioners (GPs) hesitate to engage in treating alcohol problems due to time constraints and lack of knowledge. Screening and brief interventions are effective for high consumers but there are few studies on dependence.

**Methods:**

This is a two-group, parallel, randomized controlled trial (RCT). The aim is to study whether an Internet-based Cognitive Behavioral Treatment (iCBT) when added to treatment as usual (TAU) is more effective than TAU only for alcohol dependence in PC. Two hundred and sixty adults with alcohol dependence will be included. Participants are randomized to iCBT and TAU or TAU only. The primary study outcome is alcohol consumption in grams per week and heavy-drinking days. Secondary outcomes include alcohol-related problem severity, number of diagnostic criteria for alcohol dependence, depression and anxiety symptoms, health-related quality of life and biochemical markers for high consumption and liver pathology. Data will be analyzed using mixed-effect models.

**Discussion:**

Internet-based interventions are attractive to, and have been shown to reach, people with alcohol problems. Yet there are no studies investigating the efficacy of Internet treatment of alcohol dependence in PC. In this study we hypothesize that iCBT when added to TAU will improve treatment outcome for alcohol dependence in PC, compared to TAU only. If effective, iCBT can be distributed to the public to a low cost for a stakeholder and has the opportunity to reduce both short-term and long-term public health costs.

**Trial registration:**

ISRCTN69957414. Retrospectively registered on 7 June 2018.

## Background

Alcohol dependence is a common disorder, leading to much suffering for the individuals as well as their families and the community. In a recent systematic analysis for the Global Burden of Disease Study 2016, alcohol is found to be a leading risk factor for global disease burden and causes substantial health loss [1]. It was found that the risk of all-cause mortality, and of cancers specifically, rises with increasing levels of consumption. The level of consumption that minimizes health loss is zero. Based on the net burden, 1 in 7 deaths in men and 1 in 13 deaths in women in the EU are estimated to be caused by alcohol consumption [2]. Alcohol-use disorders (AUD) have the second highest burden of disease of all mental disorders after depression, the highest in men [3].

A major problem is that most individuals with alcohol dependence are not reached with treatment. Alcohol dependence has the widest treatment gap between the number of individuals affected and the number in treatment [4]. Only around 10–20% seek help and treatment-seekers tend to have more severe dependence with co-morbid disorders and unstable social situation [5, 6]. Early interventions and formal treatment for AUD should be implemented to reduce high levels of adverse outcomes.

In previous studies from our group it was observed that most alcohol-dependent persons have a moderate level of dependence and live under socially orderly conditions [7]. Nevertheless, the main part of morbidity and mortality as well as community costs related to alcohol consumption occur in this large group with moderate dependence. The majority of affected individuals are reluctant to seek treatment in existing addiction care units, largely due to the stigma related to alcohol problems and treatment [8]. An aim for this project is to study outcomes from treatment in a context that is perceived as less stigmatizing.

One possible way of reducing stigma is treatment in primary care (PC). Studies indicate that people with AUD are positive to seeking treatment in primary care [8]. Heavy alcohol use causes or complicates many diseases and conditions and is consequently relevant to discuss in many PC consultations [9]. In addition, a large proportion of individuals with alcohol dependence are already present in PC for the treatment of other conditions [4]. Previous positive studies in PC have mostly involved hazardous drinkers and it is unclear whether dependent individuals have been included in the studies [10]. General practitioners (GPs) are at present reluctant to engage in this area, mostly due to time constraints and uncertainty regarding their competence in this field [11]. However, in a previous study on treating alcohol dependence within PC, the GPs reported that it was less complicated than anticipated to treat alcohol dependence [12]. In this study a program for brief intervention and pharmaceutical treatment tailored to the primary care setting for treating alcohol dependence was developed. Participants were randomized to treatment in PC or an addiction clinic. In this study treatment in specialized care was not superior to PC [13].

Internet-based interventions have been shown to be attractive for, and to reach, persons with alcohol problems [14]. Internet-based treatments also have the advantage of high availability with the potential of attracting participants who would otherwise not participate in treatment due to practical concerns or time constrains [15]. Interventions can be delivered to many persons simultaneously regardless of the time of the day and geographical location, may be cost-effective and reduces stigma [16]. Most Internet studies so far have involved hazardous drinkers in student or general populations [17]. Internet-based approaches have shown similar efficacy as face-to-face treatment [18]. Web-based advice to reduce drinking among hazardous drinkers has been successfully implemented in PC in a project in Italy [19].

To date, there are no randomized controlled trials of Internet-based approaches for alcohol-dependent patients in PC.

### Aims and research hypothesis

The overall aim of this project is to study new approaches to improve the identification and treatment of alcohol dependence in PC. Specifically, the aim is to investigate the efficacy of an Internet-based CBT program for alcohol-dependent patients in PC.

The research hypothesis for the project is that:

Internet-based Cognitive Behavioral Treatment (iCBT), when added to treatment as usual (TAU) for alcohol dependence in PC, will reduce alcohol consumption more than TAU only.

## Methods

**Fig. 1:**
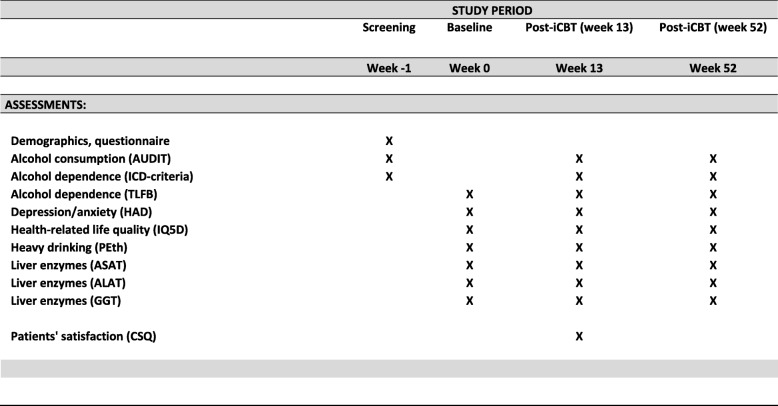
Standard Protocol Items: Recommendations for Interventional Trials (SPIRIT) Figure

We adhered to the Standard Protocol Items Recommendations for Interventional Trials (SPIRIT) guidelines in the preparation of this protocol (see Fig. 1 and Additional file 1 for the SPIRIT Figure and Checklist, respectively).

### Setting and participants

Project coordination will take place at “Riddargatan 1: Clinic for Alcohol and Health”, an outpatient alcohol clinic located in Stockholm specializing in treating AUDs and part of the Stockholm Center for Dependency Disorders. We collaborate with a number of PC clinics within Stockholm County in the study. Participants are informed about the study through the leaflet “Feel better with less alcohol”, distributed at the registration desk of the PC study sites to all patients > 18 years of age over a time period. Patients are also recruited via advertisements placed in waiting rooms at the participating PC clinics and via advertisements on the Internet and in local newspapers. Participants must be listed on and have their GP in one of the collaborating PC clinics.

#### Inclusion criteria

Participants have to meet criteria for alcohol dependence, based on the *International Statistical Classification of Diseases and Related Health Problems – Tenth Revision* (*ICD-10*). Participants also have to meet the threshold for hazardous drinking, which is defined as six or more points for women and eight or more points for men on the Alcohol Use Disorders Identification Test (AUDIT) [20].

#### Exclusion criteria

Individuals with serious mental illness, including substance-use disorder other than alcohol or nicotine, persons deemed in need of specialist treatment in psychiatry or dependence care, cognitive impairment and lack of knowledge of the Swedish language will be excluded.

#### The web-based treatment platform

We have built a web-based treatment platform with questionnaires for initial assessment and follow-up and a series of treatment modules based on cognitive behavioral therapy (CBT) and motivational enhancement therapy, here called Internet-based Cognitive Behavioral Treatment (iCBT). Prior to starting the randomized controlled trial (RCT), a pilot study (*n* = 3) was conducted involving an intervention identical to that used in the present study. The purpose was to test the study protocol, the Internet assessment procedure and the web-based treatment platform to obtain feedback from the patients on the feasibility.

### Study design

**Fig. 2:**
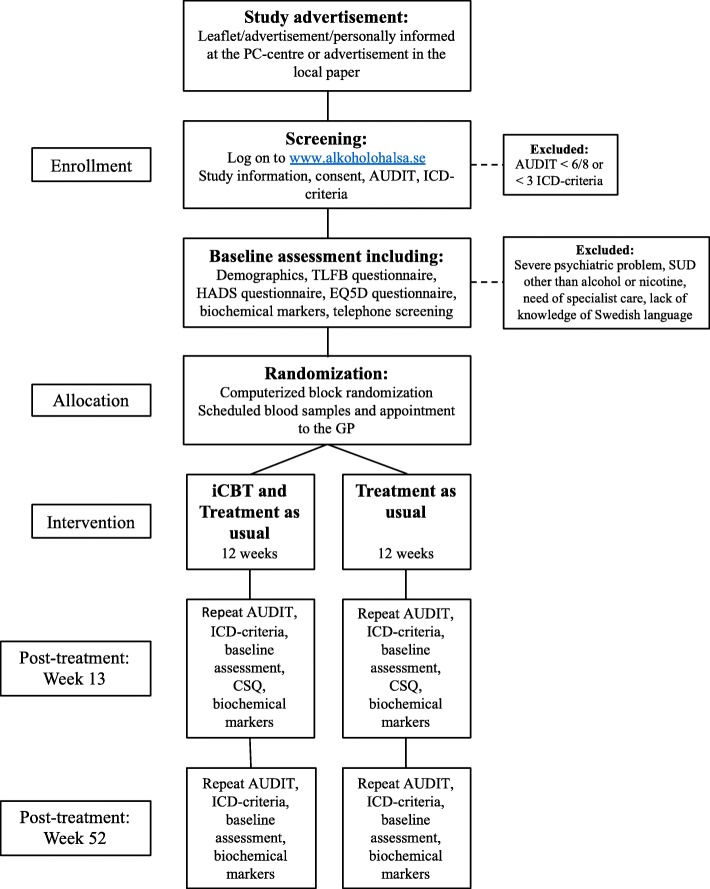
Participant flow diagram

This is a multi-center, two-group, parallel, RCT to compare the effects of two 12-week interventions: Internet-based Cognitive Behavioral Treatment (iCBT) plus treatment as usual (TAU) or TAU only, designed to demonstrate statistical superiority. The proposed flow of participants through the trial is shown in Fig. 2. Assessments and biomarkers are completed by all participants at baseline (pre randomization) and post treatment at week 13 and week 52.

### Procedure

#### Recruitment

The personnel at the PC clinics are asked to encourage all patients attending the PC clinic under the time period to log on to the website indicated on the leaflet. Individuals interested in participating click on a link to the study registration site. Here the participants are provided with information about the study, they sign an informed consent, leave their data on demographics and contact information. Participants provide an e-mail address for communication.

#### Screening and baseline assessment

Initially a web-based screening assessment takes place. Individuals whot fulfill the inclusion criteria (alcohol dependence and hazardous consumption) are automatically invited to complete baseline assessment. Individuals that do not fulfill the inclusion criteria are excluded and guided to where to get further help.

After completion of self-assessment on the website an extended telephone screening with a research nurse takes place to assure data quality and completeness. In the telephone conversation the research nurse goes through the exclusion criteria. Individuals that meet the exclusion criteria are advised to seek appropriate action.

#### Randomization, treatment allocation and blinding

The randomization was done in blocks of 20 according to a fully automated and concealed procedure in the online platform that was programmed in the content management system Drupal (drupal.org) by the fourth author. The research nurse randomizes all eligible participants via the online platform by activating a link that only she has access to. Type of treatment (iCBT + TAU vs TAU) is not blinded neither for the participants nor the research nurse. Hence, unblinding of participants and the research nurse will not be necessary during the trial. Responsible researchers as well as data analysts will not have access to the codes concerning which experimental condition the participants participate in until after completion of analysis of the primary outcome measure. Hence, the risk of bias in the interpretation of the study’s result is minimal.

The participants are informed by the research nurse about their group allocation and are scheduled for a blood test for biomarkers and an appointment with the GP at the PC clinic, ideally within 2–3 weeks. The GPs give all participants feedback on the assessment and biomarkers and design a treatment plan based on current routines at the PC clinic. For those randomized to iCBT, the Internet program will be made available the following Monday after randomization.

#### Treatment as usual (TAU)

The GPs design a treatment plan based on current routines for treating alcohol problems at the PC clinic. The fact that all participants are offered contact with a GP enables participants in need of specialized care of any kind to be referred by the GP. All GPs are offered a brief training in giving feedback on the assessment and biomarkers and the use of medication for treatment of alcohol dependence. Together this serves as the base of TAU which is provided to the participants in both study arms.

#### Internet-based Cognitive Behavioral Therapy

The intervention group will be given iCBT in addition to TAU. The iCBT program is based on the self-help material used in previous studies on the Internet [18, 21] and in primary care [13]. iCBT includes five elements: (1) Motivation to cut down or stop drinking, (2) Goal-setting for alcohol consumption per occasion and weekly consumption and self-control strategies; for example, not to have alcohol at home and strategies to drink more slowly, (3) Chart risk situations and conduct behavior analysis on occasions with too much alcohol, (4) Plan alternative activities to drinking alcohol and (5) Relapse prevention and crisis plan and mapping of life situations or stressors that can lead to relapse. The intervention is open-ended, meaning that participants can log on as often and for as long as they want.

For each assignment an informational text is provided and a home assignment is included. iCBT is an extended self-help intervention with automated feedback where participants repeatedly are reminded to start and complete every assignment.

#### Outcome measures

Primary outcome is change in alcohol consumption in mean grams per week and mean number of days with heavy drinking (that is > 4/5 drinks for women and men) the last 30 days from baseline to 12 and 52 weeks post inclusion. A drink contains 12 g of alcohol.

For all secondary outcomes mean change from baseline to 12 and 52 weeks post inclusion applies; hazardous drinking and severity of dependence, health-related quality of life, symptoms of depression and anxiety and difference in biomarker levels of heavy drinking and liver pathology.

#### Baseline and follow-up assessments

Questionnaires: Time Line Follow Back 30 days [﻿20﻿] is used to assess mean weekly alcohol consumption and heavy-drinking days. Severity of hazardous drinking and dependence is assessed with the Alcohol Use Disorders Identification Test (AUDIT) [22] and the *ICD-10* criteria for alcohol dependence (The World Health Organization, 1992). Symptoms of anxiety and depression are assessed with the Hospital Anxiety and Depression Scale (HADS) [22]. The EuroQol five dimensions, five levels health survey (EQ-5D-5 L) questionnaire measure health-related quality of life [23]. Satisfaction with treatment is assessed, only at 12 weeks’ follow up, with the Client Satisfaction Questionnaire (CSQ) [24].

Biomarkers: blood is analyzed for standard biomarkers of heavy drinking and liver pathology at each assessment; levels of phosphatidylethanol (PEth); gamma-glutamyl transferase (GGT); aspartate amino transferase (AST) and alanine amino transferase (ALT).

Biological specimens will not be stored as a part of the study. Results from blood samples are provided via the patient file system at the PC clinics and blood samples are destroyed after analysis.

Patients will be contacted by automatically created messages in the web-based treatment platform and by the research nurse when necessary during weeks 13 and 52 in order to conduct the follow-up assessments and blood tests. At week 13 the Client Satisfaction Questionnaire (CSQ) is added. Assessment completeness is checked automatically on the treatment platform and if not completed participants cannot proceed to the next step. Participants will be contacted by the research nurse and reminded if assessments are not completed using a combination of messages from the treatment platform, phone calls, emails and text messaging in order to improve retention in treatment and prevent loss to follow-up over the follow-up period. The research nurse will also remind the participants to leave the blood tests during weeks 13 and 52 and collect the results from the patient files.

If considerable somatic or psychiatric complications occur during the study, the research personnel or the GPs can take the initiative to withdraw the patient from the study and if applicable make a referral to an appropriate care-giver. Following completion of the study no post-trial care is planned as part of the study, but since all patients are in contact with their GPs further treatment may be provided within the framework of TAU.

#### Program engagement

Program engagement will be measured by automatic data collection on number of assignments accessed, tasks completed. Association between program engagement metrics and treatment outcomes will be explored.

### Statistical analyses and sample size calculations

Baseline participant characteristics stratified by treatment group will be presented. Group differences will be tested using a *t* test for continuous variables and chi-square tests for categorical outcomes. Multiple imputation will be used to replace missing internal values where appropriate. Within-group changes in the primary outcome (alcohol consumption) and secondary outcomes (*ICD-10* criteria, AUDIT scores, depression, anxiety, EQ-5D-5 L and biomarkers) at 12 and 52 weeks will be analyzed according to the intention-to-treat principle, using linear mixed models and Poisson regression where appropriate. The models will further be adjusted for baseline covariates and treatment adherence. Per-protocol analyses will also be presented. All tests will be two-sided and *p* values of less than 0.05 considered statistically significant.

The study is designed to demonstrate statistical superiority. An effect-size of 0.61 has been shown in previous Internet studies on high consumers in the general population [25]. Due to a lack of studies on alcohol-dependent patients, our estimate is based on a more conservative effect-size of 0.4, which requires the estimated sample size of 100 participants in each arm to get a power of 80% and a statistically significance 0.05 when using a *t* test. Given an estimated dropout in iCBT of 30%, 260 participants are included in the study [26].

No interim analyses are planned.

### Patient and public involvement

The need for the investigation of the efficacy of the iCBT intervention was informed by clinical experience and by previous research conducted within our research group. We collected information from GPs on how to perform the iCBT intervention with as little extra workload for the GPs as possible, when planning the study. We also conduct semi-structured interviews with a number of the GPs involved in this study on factors that can facilitate and hamper implementation of iCBT in primary care, results which will be published in a separate paper. Patients were not involved in the design or execution of the trial. Participants will have the possibility to access their individual study data through the intervention platform. Results from the study will be disseminated through journal articles, conference presentations and reports to the funding agencies. The burden of the intervention was not assessed by patients prior to commencing the trial; patients’ satisfaction with the treatment in the study will, however, be collected as part of the post-intervention assessment.

## Discussion

This study protocol describes the first study on the efficacy of an Internet cognitive behavioral intervention for alcohol dependence in PC. The intervention program evaluated in this randomized controlled superiority trial is designed to be accessible and easy to use. Another purpose when designing this intervention program has been to add as little workload as possible for the GPs to refer the patients to treatment. This intervention can be made available to the public at a low cost for a stakeholder.

Treatment in PC and Internet interventions are less stigmatizing and are seen as more appealing than specialized care in this group. Unless PC is equipped with tools to treat patients with hazardous alcohol consumption and mild and moderate dependence, GPs might continue to hesitate to raise questions about alcohol. By offering evidence-based psychological and pharmacological treatment, it is possible to treat the large majority of dependent patients with low to moderate levels of dependence. If this iCBT program, as we hypothesize, contributes to a better treatment outcome it provides PC with a new tool to assist more patients with drinking problems. Thereby the health burden related to alcohol-use disorders may be reduced. It may also decrease the reluctance that practitioners feel to raise the subject of alcohol and the threshold for individuals to seek help can be lowered. In this study the main benefit is that all participants are offered treatment (TAU) for their substance-use disorders, treatment that otherwise in most cases would not have taken place. Even patients in the control group will receive interventions which are more extensive than otherwise. That is, feedback on assessments, brief advice and offers of pharmacological treatment. A widespread distribution of the intervention is possible and has the opportunity to reduce both short-term and long-term public health costs. Making treatment more available and appealing to the whole population is also an important contribution to increased health equality.

### Trial status

The recruitment commenced in September 2017 and will be completed in December 2019. The study will conclude in December 2020. This is the first protocol version. Issue date: 5 June 2019.

## Supplementary information

**Additional file 1.** Standard Protocol Items: Recommendations for Interventional Trials (SPIRIT) 2013 Checklist: recommended items to address in a clinical trial protocol and related documents.

## Data Availability

During the trial, all patient data will be kept in a secure room accessible only by the research group, following standard protocols recommended by the Karolinska Institutet. Participant-level data and statistical codes are available on reasonable request according to the regulations of the responsible organization. All data are stored in a secure database available only to the research team. An external data monitoring committee or auditing were not deemed necessary due to the minimal risks involved, because data registration is done automatically in the online platform where all data are stored. The online platform is maintained and updated weekly. Data from blood samples are collected directly from the patient data file system at the PC clinics. All the data management procedures were monitored by the author group.

## Abbreviations

AUD: Alcohol-use disorders
GP: General practitioner
iCBT: Internet-based Cognitive Behavioral Treatment
PC: Primary care
RCT: Randomized controlled trial
TAU: Treatment as usual
